# Contribution of interaction force to the sense of hand ownership and the sense of hand agency

**DOI:** 10.1038/s41598-021-97540-9

**Published:** 2021-09-10

**Authors:** Michel Akselrod, Bogdan Vigaru, Julio Duenas, Roberto Martuzzi, James Sulzer, Andrea Serino, Olaf Blanke, Roger Gassert

**Affiliations:** 1 Rehabilitation Engineering Laboratory, Department of Health Sciences and Technology, ETH Zurich, Zurich, Switzerland; 2 Department of Clinical Neuroscience, MySpace Laboratory, University Hospital of Lausanne (CHUV), Avenue Beaumont, Pavillon 4, 1011 Lausanne, Switzerland; 3 Cognition, Motion and Neuroscience Unit, Fondazione Istituto Italiano Di Tecnologia, Minded Programme, Genova, Italy; 4 Laboratory of Cognitive Neuroscience, Federal Institute of Technology of Lausanne (EPFL), Lausanne, Switzerland; 5 Fondation Campus Biotech Geneva, Geneva, Switzerland; 6 Rewire Laboratory, University of Texas at Austin, Austin, USA; 7 Department of Neurology, University Hospital of Geneva, Geneva, Switzerland

**Keywords:** Cognitive neuroscience, Human behaviour

## Abstract

When performing willed actions, we have the unified and coherent experience of owning and controlling our body. Body ownership is believed to emerge from the integration of coherent multisensory signals, while agency is believed to emerge from the coherence between predicted and perceived outcomes of actions. As a consequence, body ownership and agency can both be modulated by multisensory conflicts. The contribution of active movement generation to ownership and agency has not been parametrically explored. Here, we investigated the contribution of interaction force between the agent and the environment to the sense of hand ownership (SO) and the sense of hand agency (SA). By combining robotics and virtual reality, we manipulated the sensorimotor and visual information during immersive scenarios to induce and quantify altered states of SO and SA. First, we demonstrated that SO and SA could be successfully manipulated by our experimental paradigms. Second, we showed that interaction force strongly contributes to SA, but to a lesser extent to SO. Finally, we showed that SO and SA interact beyond their common multisensory basis. Our results, based on two independent studies, provide a direct link between sensorimotor interactions and subjective body experience and demonstrate a new dissociation between SO and SA.

## Introduction

Beyond sensing and moving our hands, the experience that our hands “belong” to us and that we are in control of their actions is a complex and natural phenomenon that accompanies hand actions under normal conditions. The sense of hand ownership (SO) is the subjective experience that the hand is identified with the self ("this is my hand"), and the sense of hand agency (SA) is the subjective experience that the self is identified as the agent of the hand's actions ("I am controlling this hand")^1–3^. The experimental manipulation of multisensory and sensorimotor signals has been extensively used to study the mechanisms associated with SO and SA. In particular, seeing an external hand performing an action (e.g. a fake or virtual hand), which is compatible with the action of one’s own hand, will induce SO and SA towards the external hand. Several factors will influence how strongly SO and SA are experienced towards the seen hand, including the compatibility of visual appearance and movement dynamics between the seen hand and one’s own hand^4–15^.

The primary goal of movements of the upper extremity is to dynamically interact with the environment, e.g. to manipulate objects, which generates rich somatosensory feedback. Object manipulation requires an adaptive control of the exerted force and relies on the perception of force feedback. The interaction force between the agent and the environment (e.g. a manipulated object) is processed at a low-level for efficient motor control. However, no study has explored whether interaction force is also involved in the mechanisms associated with SO and SA. The present study aims at investigating the respective contributions of interaction force between the agent and the environment to SO and SA. This is an important research topic to better understand the link between motor control, sensory feedback and subjective body experience (e.g. SO and SA), but is also relevant for the fields of robotics and neuroprosthetics, which aim at developing devices that can be interfaced with the nervous system and are perceived as integral parts of the body.

SO and SA are classically measured with subjective reports from the participants who indicate on a Likert scale whether they experience the external hand as their own (SO) or whether they feel in control of the external hand (SA). These subjective measures have been largely validated in the literature^4–15^. In addition, objective measures to quantify the strength of the illusion have also been used, such as the proprioceptive drift^16^. It has been shown that when SO and SA are experienced towards an external hand, participants localize the perceived position of their own hand shifted towards the seen external hand, indicating that a proprioceptive drift has occurred^4,8,9,13–15^. The proprioceptive drift is considered as an objective measure of the strength of the illusion, however, subjective reports and the proprioceptive drift do not necessarily correlate^17–20^, suggesting that these measures capture different aspects of the illusion.

Previous studies have investigated the experimental alteration of SO and SA, as well as their interplay. SO is classically manipulated by the temporal synchrony between multisensory signals^16^ or by the congruent structure of the owned hand (e.g. shape, position or orientation). Contrastingly, SA can be altered by the temporal synchrony between sensorimotor signals and requires an active movement production of the agent^5,6,9–11,14,21^**.** An important distinction between SO and SA is that SO can be manipulated under static conditions, while the presence of movements seems necessary to manipulate SA. This suggests a partial dissociation between SO and SA. In addition, several studies reported a strong correlation between SO and SA suggesting a certain degree of interaction between the two^6,10,11,21^. Other studies suggested that SA enhances and extends SO, thus further supporting possible interactions between SO and SA^4,7,8,13,15^. Importantly, SO and SA are modulated by the same experimental factors (e.g. synchrony between felt and seen movements), thus their interplay might rely on their common multisensory basis and no study has so far thoroughly accounted for this confound. In addition, motor attributes of movements might contribute differently to SO and SA, but only few studies investigated such aspects^14,22–24^, and no study has parametrically explored interaction forces between the agent and the environment and their respective contributions to SO and SA.

Studying the contributions of interaction force to SO and SA is methodologically challenging^4,6–8,10,11,13,15,21^. Indeed, it requires the simultaneous capture of movements of the agent’s hand, the dependent control of movements of the seen hand, and the measure of associated interaction force. With the advent of virtual reality, it is possible to manipulate visual information in unprecedented ways, thus improving the control over experimental stimuli. Using virtual reality, studies successfully induced SO or SA towards virtual hands^12,13,22,25–34^. Furthermore, the combination of virtual reality and robotics allows manipulating the visual and sensorimotor (i.e. tactile, proprioceptive and motor) information in a controlled and reproducible manner. In particular, the use of robotic devices allows investigating the respective contributions of passive and active movements to SO and SA, and to measure interaction force between the agent and the robotic device using force sensors.

The goals of the present study are twofold. The first goal is to investigate the contribution of interaction force to SO and SA and whether this force interacts with other known modulators of SO and SA. The second goal is to determine whether SO and SA are still correlated even when accounting for their common multisensory basis (i.e. the shared variance associated with their common modulators). To this end, we developed novel experimental setups combining robotics and virtual reality and conducted two independent studies with identical designs for replicative purposes (n = 27 for Study I and n = 26 for Study II). Using an immersive VR scenario, we manipulated the coherence between visual and sensorimotor information to induce altered states of SO and SA. With a 2 × 2  × 2 design, we manipulated 3 experimental factors: 1) "movement type" (active or passive movement); 2) “synchrony” (synchronous or asynchronous movement of the virtual hand with respect to the real hand); and 3) “congruency” (right or left virtual hand moving). During each trial, we measured the participants’ subjective ratings of SO and SA towards the virtual avatar’s hand, as well as the interaction force between the participant’s hands and the robotic device. To investigate the contribution of interaction force to SO and SA, we used linear mixed models and analyzed SO and SA ratings as modeled by the 3 experimental factors (movement type, synchrony and congruency) and the force as covariate. We hypothesized that interaction force will have a strong impact on SA due to the role of interaction force in motor control and efferent mechanisms, and less impact on SO, which is considered a rather perceptual phenomenon. In addition, we analyzed the correlation between SO and SA while accounting for their shared variance associated with the experimental factors. We expected a strong relationship between SO and SA even after regressing out the shared variance explained by the experimental factors. These data are relevant for better understanding the relationship between motor control, sensory feedback and subjective body experience (e.g. SO and SA), and might translate into novel strategies to facilitate user-device interactions for the fields of robotics and neuroprosthetics.

## Results

### Ownership ratings modelled by experimental factors and interaction force

#### Study I

**Figure 1:**
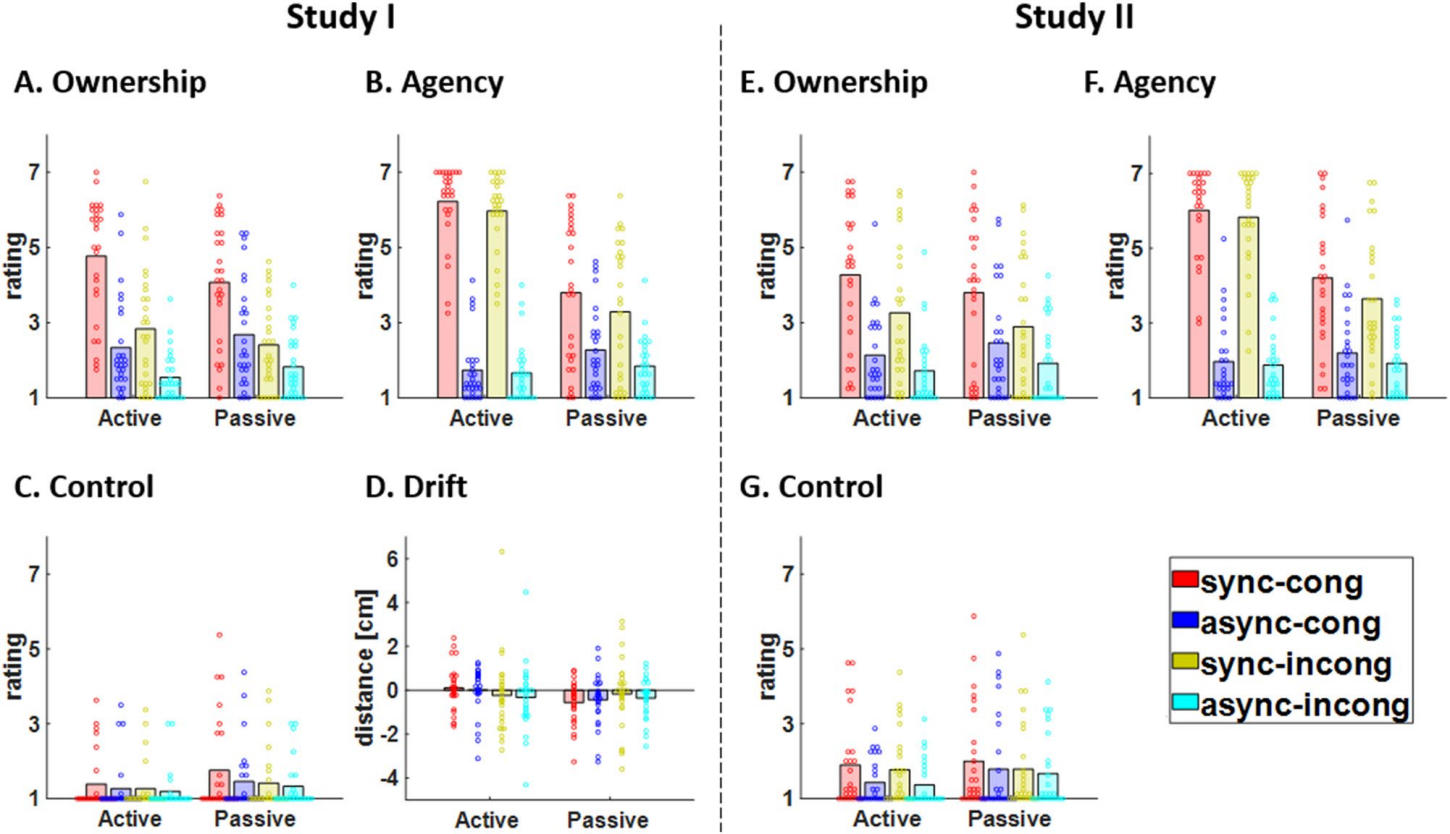
Questionnaire results. (**A**) Average raw SO ratings for Study I, rated from 1 (least SO) to 7 (greatest SO). (**B**) Average raw SA ratings for Study I. (**C**) Average raw control ratings for Study I. (**D**) Average proprioceptive drift for Study I. (**E**) Average raw SO ratings for Study II. (**F**) Average raw SA ratings for Study II. (**G**) Average raw control ratings for Study II. For all results, the 8 experimental conditions follow a 2 × 2  × 2 design: active/passive x synchronous/asynchronous x congruent/incongruent.

**Figure 2:**
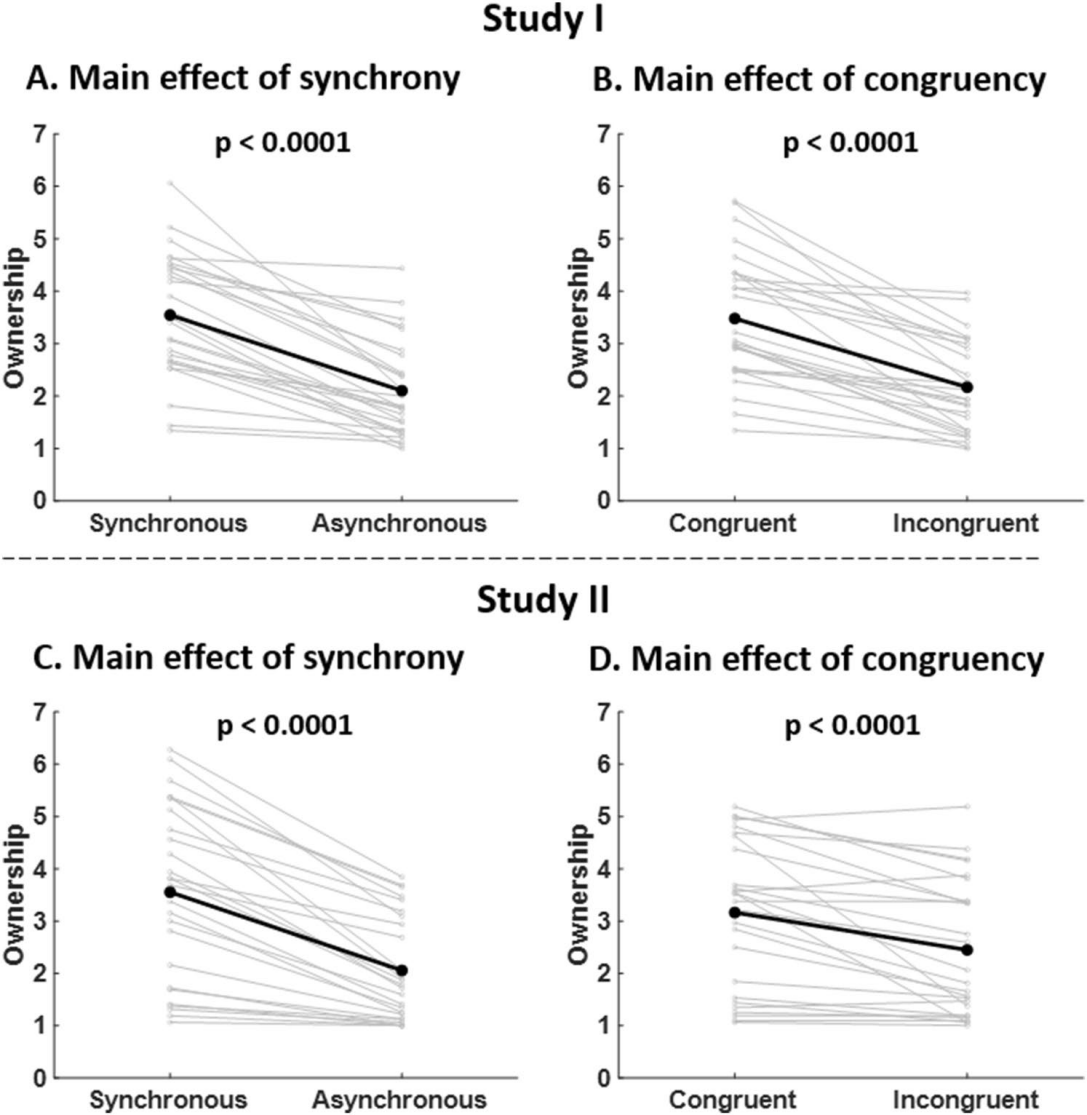
SO ratings (Studies I and II). (**A**–**C**) SO ratings for synchronous and asynchronous conditions are shown for Studies I and II. (**B–D**) SO ratings for congruent and incongruent conditions are shown for Studies I and II. For each panel, data plotted in grey represent the mean rating for individual subjects and data plotted in black represents the mean rating across subjects. The p-values correspond to the main effects reported in Table 1.

Analyzing subjective ratings of SO ("I felt as if the virtual hand was my own hand", Fig. 1A), we found a main effect of "synchrony" (t_1653_ = 13.28, *P* < 0.0001, Table 1). This effect was explained by greater SO ratings during synchronous conditions compared to asynchronous conditions (mean difference, MD = 1.44, standard deviation, SD = 0.86, Fig. 2A). We also found a main effect of "congruency" (t_1653_ = 13.21, *P* < 0.0001, Table 1). This effect was due to greater SO ratings during congruent conditions compared to incongruent conditions (MD = 1.31, SD = 0.85, Fig. 2B). We note that we did not find a main effect of movement type, nor a main effect of force, nor an interaction involving the factor of force (Table 1), suggesting that in Study I SO is not modulated by the type of movement and that SO is not modulated by the force.

**Table 1:** Statistical results for Study I.

Effect	Ownership			Agency			Control			Drift		
	d.o.f	T-statistic	*P*-value	d.o.f	T-statistic	*P*-value	d.o.f	T-statistic	*P*-value	d.o.f	T-statistic	*P*-value
MOVE	1680	1.04	0.30	1667	7.65	< 0.0001	1668	− 4.95	< 0.0001	529	− 0.46	0.65
SYNC	1653	13.28	< 0.0001	1653	14.64	< 0.0001	1653	2.89	0.004	1656	0.64	0.52
CONG	1653	13.21	< 0.0001	1653	4.32	< 0.0001	1653	3.37	0.0008	1653	− 0.55	0.58
FORCE	1679	− 1.19	0.23	1675	− 0.78	0.44	1666	− 3.17	0.002	622	− 1.55	0.12
MOVE*FORCE	1680	0.34	0.73	1669	− 1.79	0.07	1667	2.98	0.003	526	1.46	0.15
SYNC*FORCE	1654	1.86	0.06	1654	18.57	< 0.0001	1653	0.18	0.85	1662	− 0.62	0.54
CONG*FORCE	1653	0.47	0.64	1653	− 1.78	0.08	1653	0.07	0.95	1653	1.12	0.26

SO ratings, SA ratings, control ratings and proprioceptive drifts (Study I only) are modelled as a function of the experimental factors ("movement type", "synchrony" and "congruency") and the "force”.

#### Study II

Subjective ratings of SO ("I felt as if the virtual hand was my own hand", Fig. 1E) showed a main effect of "synchrony" (t_1639_ = 9.86, *P* < 0.0001, Table 2). This effect was explained by greater SO ratings during synchronous conditions compared to asynchronous conditions (MD = 1.50, SD = 0.95, Fig. 2C). We also found a main effect of "congruency" (t_1638_ = 5.42, *P* < 0.0001, Table 2). This effect was explained by greater SO ratings during congruent conditions compared to incongruent conditions (MD = 0.72, SD = 0.84, Fig. 2D). In addition, there was an interaction "force x synchrony" (t_1639_ = 2.11, *P* = 0.04, Table 2). As in Study I, we did not find a main effect of movement type, suggesting that SO is not modulated by the type of movement.

**Table 2:** Statistical results for Study II.

Effect	Ownership			Agency			Control		
	d.o.f	T-statistic	*P*-value	d.o.f	T-statistic	*P*-value	d.o.f	T-statistic	*P*-value
MOVE	1650	0.91	0.36	1664	4.04	< 0.0001	1651	1.72	0.09
SYNC	1639	9.86	< 0.0001	1640	13.52	< 0.0001	1639	1.94	0.05
CONG	1638	5.42	< 0.0001	1638	2.48	0.01	1638	0.64	0.52
FORCE	1658	1.56	0.12	1662	− 2.30	0.02	1654	2.79	0.005
MOVE*FORCE	1653	− 1.31	0.19	1663	0.66	0.51	1653	− 3.99	0.0001
SYNC*FORCE	1639	2.11	0.04	1641	7.44	< 0.0001	1639	1.56	0.12
CONG*FORCE	1638	0.36	0.72	1638	− 0.72	0.47	1638	0.96	0.34

SO ratings, SA ratings, control ratings and proprioceptive drifts (Study I only) are modelled as a function of the experimental factors ("movement type", "synchrony" and "congruency") and the "force”.

**Figure 3:**
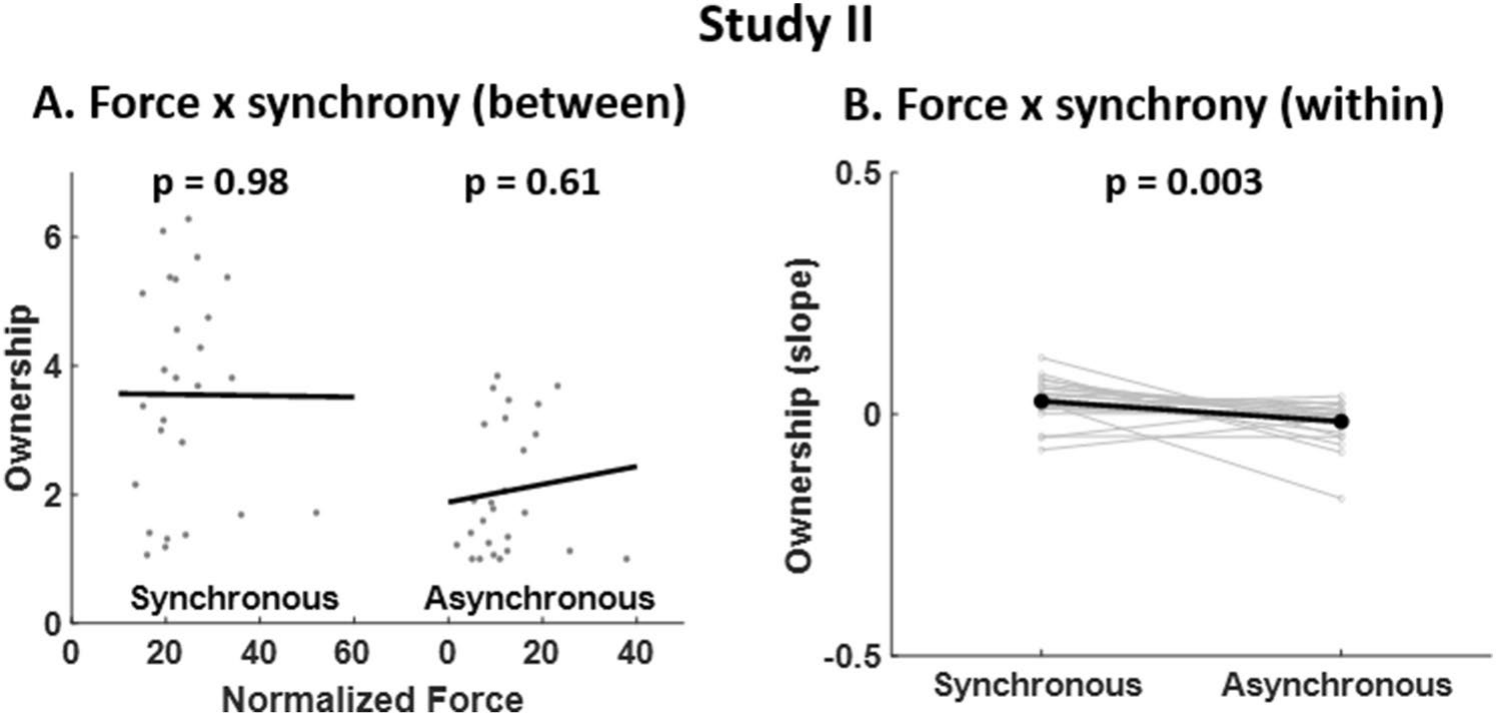
SO ratings - post-hoc comparisons for interaction “force x synchrony” (Study II). (**A**) Between-subject regressions between SO ratings and force for synchronous conditions (left) and asynchronous conditions (right) are shown. Grey data points correspond to the mean SO rating and mean force across synchronous/asynchronous trials for each subject. The black lines correspond to the between-subject regression line. The non-significant regressions suggest that the interaction “force x synchrony” (Table 1, Study II) is not explained by between-subject variability. (**B**) Within-subject regression coefficients between SO ratings and force for synchronous conditions (left) and asynchronous conditions (right) are shown. For each subject, a regression between SO ratings and force for all synchronous/asynchronous trials was computed and a paired t-test was computed between synchronous and asynchronous regression coefficients. Data points plotted in grey correspond to individual regression coefficients and data points plotted in black represent the mean coefficients across subjects. The significant paired t-test suggests that the interaction “force x synchrony” (Table 1, Study II) is explained by within-subject variability.

We further explored the respective contributions of between-subject and within-subject variability to the interaction "force x synchrony". We found that the between-subject relationship between SO and force was not significant for both synchronous and asynchronous conditions (*P* = 0.66, R^2^ = 0.01 and *P* = 0.15, R^2^ = 0.08 respectively, Fig. 3A). Instead, we found a positive within-subject relationship between SO and force for synchronous conditions (t_25_ = 3.36, *P* = 0.003). We observed a negative within-subject trend between SO and force for asynchronous conditions (t_25_ = − 1.83, *P* = 0.08), with a significant difference between asynchronous and synchronous conditions (t_25_ = 3.34, *P* = 0.003, Fig. 3B). This suggests that during synchronous conditions, an increased force leads to an increased SO, and that this effect is found within subjects across trials rather than between subjects.

### Agency ratings modelled by experimental factors and interaction force

#### Study I

**Figure 4:**
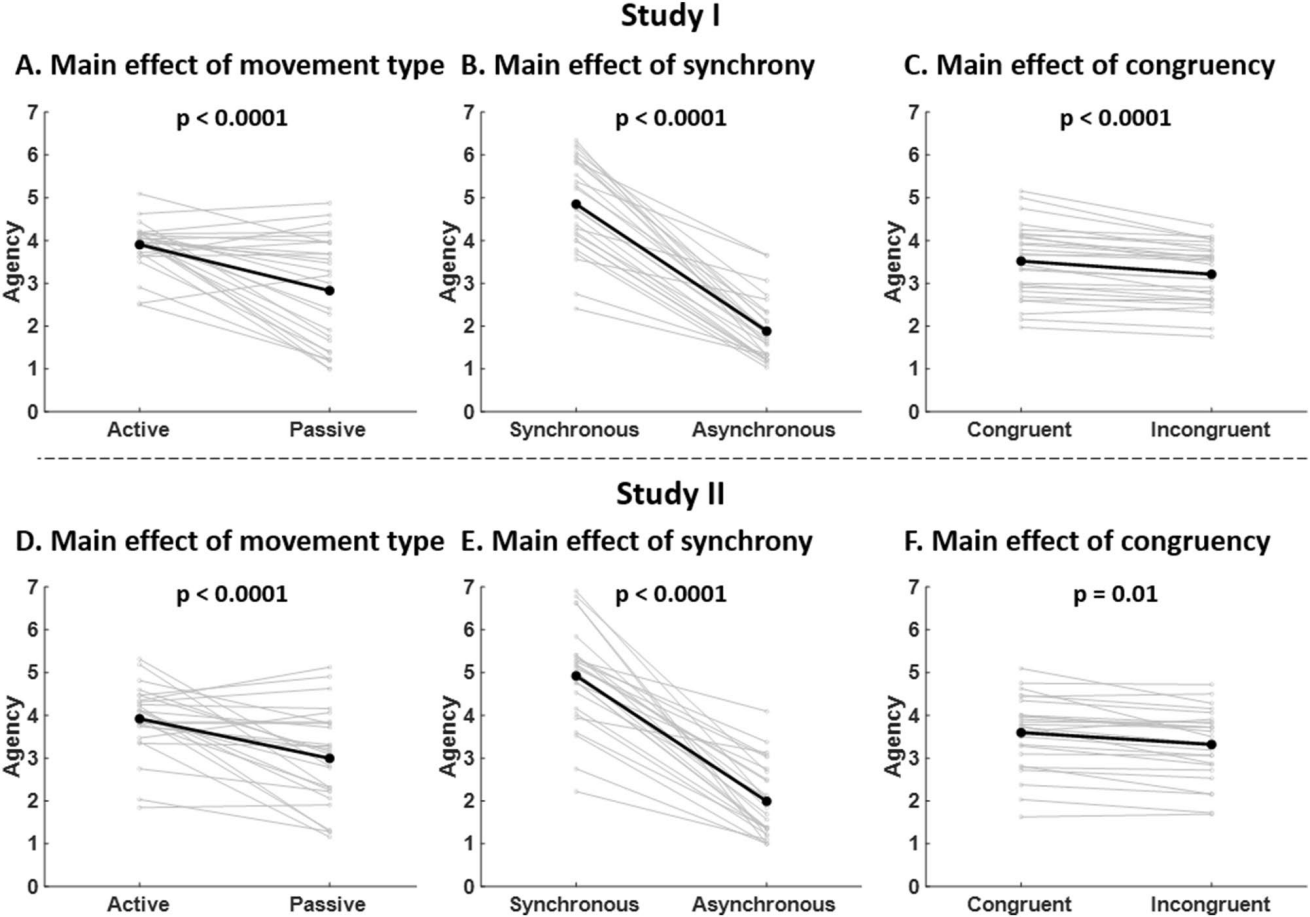
SA ratings (Studies I and II). (**A**–**D**) SA ratings for active and passive conditions are shown for Studies I and II. (**B**–**E**) SA ratings for synchronous and asynchronous conditions are shown for Studies I and II. (**C**–**F**) SA ratings for congruent and incongruent conditions are shown for Studies I and II. For each panel, data plotted in grey represent the mean rating for individual subjects and data plotted in black represents the mean rating across subjects. The p-values correspond to the main effects reported in Table 1.

For subjective ratings of SA ("I felt as if I was producing the virtual hand movements", Fig. 1B), there was a main effect of "movement type" (t_1667_ = 7.65, *P* < 0.0001, Table 1). This effect was explained by greater SA ratings during active conditions compared to passive conditions (MD = 1.08, SD = 1.21, Fig. 4A). There was also a main effect of "synchrony" (t_1653_ = 14.64, *P* < 0.0001, Table1), which was explained by greater SA ratings during synchronous conditions compared to asynchronous conditions (MD = 2.97, SD = 1.05, Fig. 4B). We also found a main effect of "congruency" (F_1653_ = 4.32, *P* < 0.0001, Table 1). This effect was due to greater SA ratings during congruent conditions compared to incongruent conditions (MD = 0.31, SD = 0.27, Fig. 4C). We also found a significant interaction "force x synchrony" (t_1654_ = 18.57, *P* < 0.0001, Table 1).

**Figure 5:**
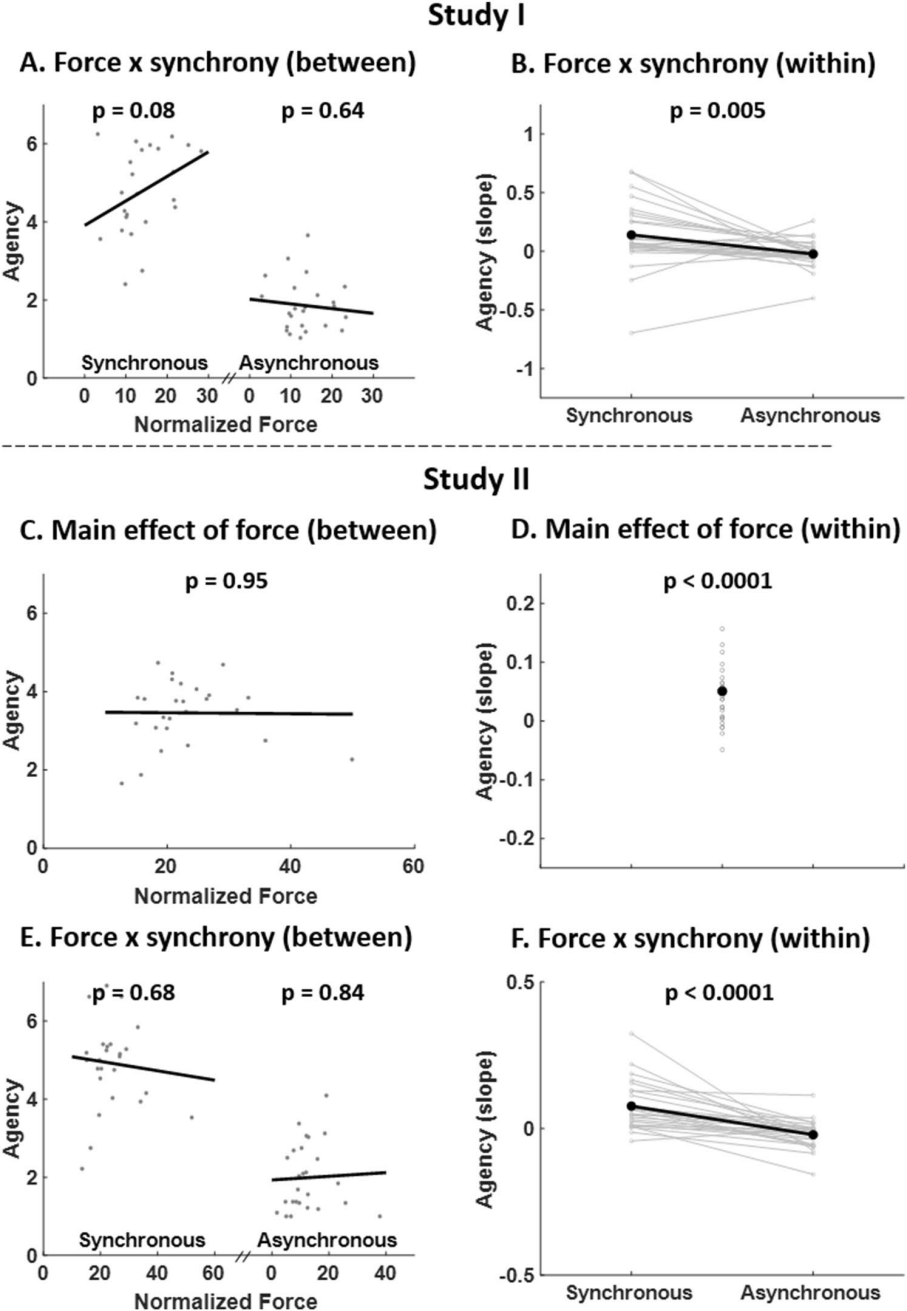
SA ratings - post-hoc comparisons for main effect of “force” and interaction “force x synchrony” (Studies I and II). (**A–E**) Between-subject regressions between SA ratings and force for synchronous conditions (left) and asynchronous conditions (right) are shown. Grey data points correspond to the mean SA rating and mean force across synchronous/asynchronous trials for each subject. The black lines correspond to the between-subject regression line. The non-significant regressions suggest that the interactions “force x synchrony” (Table 1, Studies I and II) are not explained by between-subject variability. (**B–F**) Within-subject regression coefficients between SA ratings and force for synchronous conditions (left) and asynchronous conditions (right) are shown. For each subject, a regression between SA ratings and force for all synchronous/asynchronous trials was computed and a paired t-test was computed between the synchronous and asynchronous regression coefficients. Data points plotted in grey correspond to individual regression coefficients and data points plotted in black represent the mean coefficients across subjects. The significant paired t-tests suggest that the interactions “force x synchrony” (Table 1, Studies I and II) are explained by within-subject variability. (**C**) Post-hoc comparisons as in A-E to evaluate the contribution of between-subject variability to the main effect of force. The non-significant regression suggests that the main effect of force (Table 1, Study II) is not explained by between-subject variability. (**D**) Post-hoc comparisons as in (**B**–**F**) to evaluate the contribution of within-subject variability to the main effect of force. The significant one sample t-test suggests that the main effect of force (Table 1, Study II) is explained by within-subject variability.

We further explored the respective contributions of between-subject and within-subject variability to the interaction "force x synchrony". We found that the between-subject relationship between SA and force was not significant for both synchronous and asynchronous conditions (*P* = 0.08, R^2^ = 0.13 and *P* = 0.64, R^2^ = 0.01 respectively, Fig. 5A). Instead, we found a positive within-subject relationship between SA and force for synchronous conditions (t_26_ = 2.60, *P* = 0.02) and a non-significant within-subject relationship between SA and force for asynchronous conditions (t_26_ = − 1.03, *P* = 0.31), with a significant difference between synchronous and asynchronous conditions (t_26_ = 3.06, *P* = 0.005, Fig. 5B). This suggests that during synchronous conditions, an increased force leads to an increased SA, and that this effect is found within-subjects across trials rather than between subjects.

#### Study II

Subjective ratings of SA ("I felt as if I was producing the virtual hand movements", Fig. 1F) showed a main effect of "movement type" (t_1664_ = 4.04, *P* < 0.0001, Table 2). This effect was due to greater SA ratings during active conditions compared to passive conditions (MD = 0.93, SD = 1.12, Fig. 4D). There was a main effect of "synchrony" (t_1640_ = 13.52, *P* < 0.0001, Table 2), which was explained by greater SA ratings during synchronous conditions compared to asynchronous conditions (MD = 2.93, SD = 1.24, Fig. 4E). We also found a main effect of “congruency” (t_1638_ = 2.48, *P* = 0.01, Table 2), which was due to greater SA ratings during congruent conditions compared to incongruent conditions (MD = 0.28, SD = 0.31, Fig. 4F). In addition, there was a main effect of "force" (t_1662_ = − 2.30, *P* = 0.02, Table 2) and an interaction "force x synchrony" (t_1641_ = 7.44, *P* < 0.0001, Table 2).

We further explored the respective contributions of between-subject and within-subject variability to the main effect of “force” and to the interaction “force x synchrony”. We found that the between-subject relationship between SA and force was not significant overall (*P* = 0.95, R^2^ = 0.00, Fig. 5C), nor when considering separately synchronous and asynchronous conditions (*P* = 0.68, R^2^ = 0.007 and *P* = 0.84, R^2^ = 0.001 respectively, Fig. 5E). Instead, we found a positive significant within-subject relationship between SA and force (t_25_ = 4.01, *P* = 0.0005, Fig. 5D). When considering separately synchronous and asynchronous conditions, we found a positive within-subject relationship between SA and force for synchronous conditions (t_25_ = 4.74, *P* < 0.0001) and a negative within-subject relationship between SA and force for asynchronous conditions (t_25_ = − 2.17, *P* = 0.04), with a significant difference between synchronous and asynchronous conditions (t_25_ = 5.44, *P* < 0.0001, Fig. 5F). As in study I, this suggests that during synchronous conditions, an increased force leads to an increased SA, and that this effect is found within-subjects across trials rather than between subjects.

### Ownership ratings modelled by agency ratings and interaction force

To provide a direct comparison between SO, SA and interaction force, we computed linear mixed models with SO ratings as observed variable and with SA ratings and interaction force as explanatory variables.

#### Study I

We found a main effect of SA (t_1675_ = 19.63, *P* < 0.0001, Table S1) and an interaction “SA x force” (t_1663_ = − 3.48, *P* = 0.0005, Table S1). We note that we did not find a main effect of force. In addition, the significant interaction “SA x force” suggests that force modulates SO only as a function of SA.

#### Study II

We found a main effect of SA (t_1630_ = 14.09, *P* < 0.0001, Table S1). We note that we did not find a main effect of force nor an interaction “SA x force”, which suggest no modulation of SO by the force.

### Agency ratings modelled by ownership ratings and interaction force

To provide a direct comparison between SA, SO and interaction force, we computed linear mixed models with SA ratings as observed variable and with SO ratings and interaction force as explanatory variables.

#### Study I

We found a main effect of SO (t_1630_ = 17.48, *P* < 0.0001, Table S1), a main effect of force (t_1677_ = 4.09, *P* < 0.0001, Table S1) and no interaction between the two. These results further support the previous finding that interaction force modulates SA.

#### Study II

We found a main effect of SO (t_1640_ = 14.18, *P* < 0.0001, Table S1), a main effect of force (t_1626_ = 2.63, *P* = 0.009, Table S1) and no interaction between the two. These results further support the previous finding that interaction force modulates SA.

### Control ratings modelled by experimental factors and interaction force

#### Study I

We also found significant effects for control ratings ("I felt as if my real hand was disappearing"", Fig. 1C). We found a main effect of "movement type" (t_1668_ = − 4.95, *P* < 0.0001, Table 1), which was associated with greater control ratings during passive conditions compared to active conditions (MD = 0.25, SD = 0.46, Figure S2A). We found a main effect of "synchrony" (t_1653_ = 2.89, *P* = 0.004, Table 1), which was explained by greater control ratings during synchronous conditions compared to asynchronous conditions (MD = 0.12, SD = 0.28, Figure S2B). We also found a main effect of "congruency" (t_1653_ = 3.37, *P* = 0.0008, Table1), which was due to greater control ratings during congruent conditions compared to incongruent conditions (MD = 0.13, SD = 0.30, Figure S2C). We note that the mean differences between levels of significant factors was relatively weak in comparison to the effects observed for SO and SA ratings (up to tenfold weaker). In addition, there was a main effect of "force" (t_1666_ = − 3.17, *P* = 0.002, Table 1) and an interaction "force x movement type" (t_1667_ = 2.98, *P* = 0.003, Table 1). We further explored the respective contributions of between-subject and within-subject variability to the main effect of “force”, and found no significant between-subject effect (Figure S3A) and a significant within-subject effect (t_26_ = − 2.06, *P* = 0.049, Figure S3B), which was due to lower control ratings associated with a greater force. For the interaction “force x synchrony”, we found no significant relationship between control ratings and force for between-subject (Figure S3C) and within-subject effects (Figure S3D).

#### Study II

For control ratings ("I felt as if my real hand was disappearing"", Fig. 1G). We found a main effect of "force" (t_1654_ = 2.79, *P* = 0.005, Table 2) and an interaction "force x movement type" (t_1653_ = − 3.99, *P* = 0.0003, Table 2). We further explored the respective contributions of between-subject and within-subject variability to the main effect of “force” and to the interaction “force x synchrony” and found no significant relationship between control ratings and force (Figure S3E-H).

### Proprioceptive drift modeled by experimental factors and interaction force

In Study I, there was no significant effect associated with the proprioceptive drift (Fig. 1D and Table 1).

### Interaction force modeled by experimental factors

#### Study I

We also analyzed the force as observed variable with "movement type", synchrony", and "congruency" as fixed effects (Figure S4A and Table S2). As could be expected, we found a main effect of "movement type" (t_1654_ = 38.72, *P* < 0.0001, Table S2). This effect was due to greater force during active conditions compared to passive conditions (MD = 14.16, SD = 12.05, Figure S4B). We also found a main effect of "synchrony" (t_1653_ = 1.97, *P* = 0.049, Table S2), which was explained by greater force during synchronous conditions compared to asynchronous conditions (MD = 0.11, SD = 0.84, Figure S4C). We note that the effect of “synchrony” was of much lower magnitude compared to the effect of “movement type” (120-fold weaker).

#### Study II

Similarly, analysis of force data (Figure S4D) expectedly showed a main effect of "movement type" (t_1638_ = 21.97, *P* < 0.0001, Table S1). This effect was explained by greater force during active conditions compared to passive conditions (MD = 11.74, SD = 13.56, Figure S4E). We also found a main effect of "synchrony" (t_1638_ = 2.46, *P* = 0.014, Table S1), which was due to greater force during synchronous conditions compared to asynchronous conditions (MD = 1.31, SD = 1.70, Figure S4F). We note that the effect of “synchrony” was of much lower magnitude compared to the effect of “movement type” (tenfold weaker).

### Correlation between Ownership and Agency ratings

#### Study I

**Figure 6:**
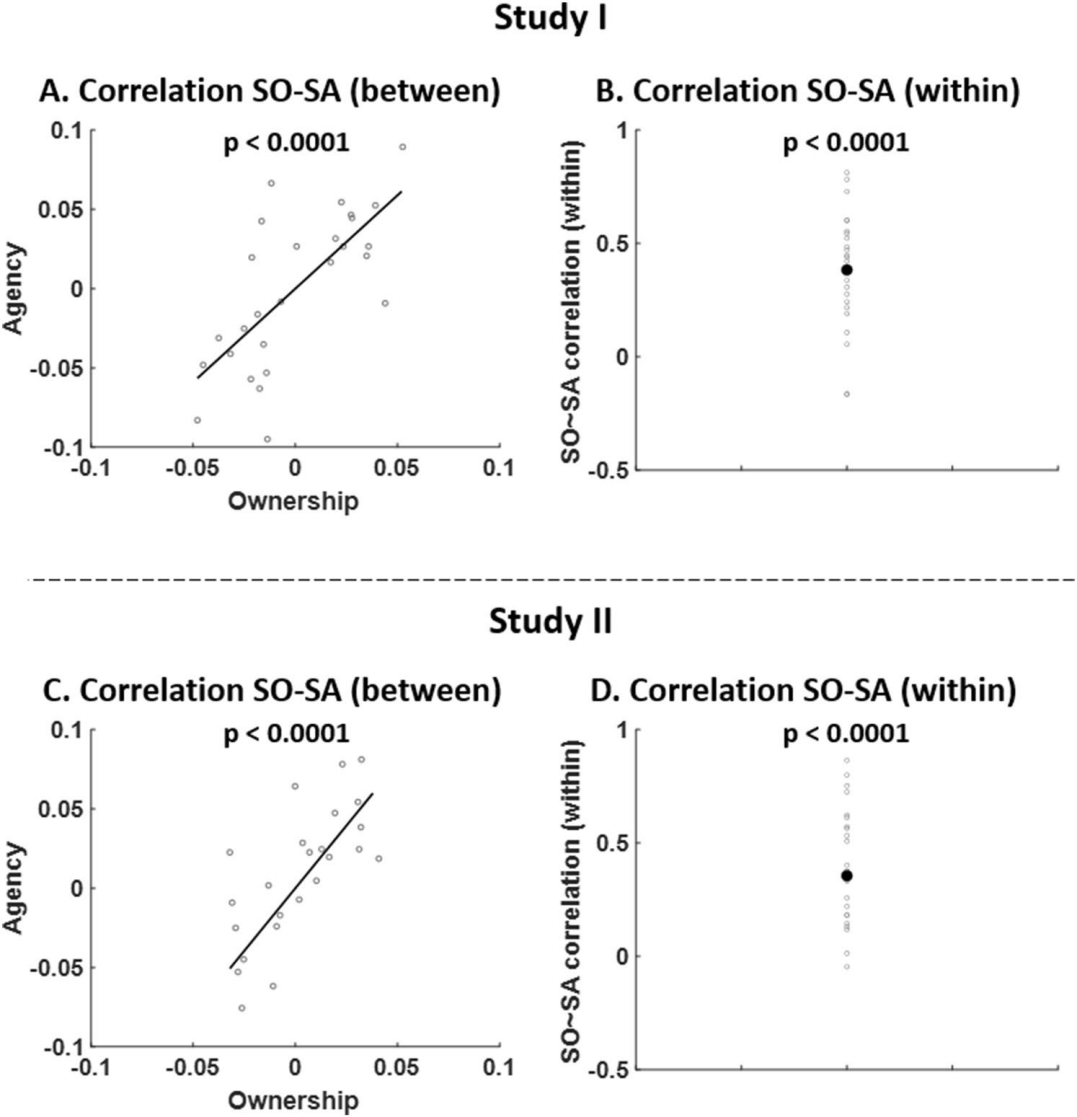
Correlation analysis. (**A**) Correlation between ownership and agency ratings for Study I. (**B**) Correlation between ownership and agency residuals for Study I. (**C**) Correlation between ownership and agency ratings for Study II. (**D**) Correlation between ownership and agency residuals for Study II.

After regressing out the variance associated with the experimental factors (“movement type”, “synchrony” and “congruency”) from SO and SA ratings, we computed correlations between SO and SA ratings both at the between-subject level and within-subject level. We found a significant positive between-subject relationship between both SO and SA (r = 0.71, *P* < 0.0001, Fig. 6A). In addition, we also found that, on a trial-by-trial basis, SO and SA correlated positively at the within-subject-level (t = 8.52, *P* < 0.0001, Fig. 6B).

We repeated the same analyses to compare SO and control ratings, as well as between SA and controls ratings. We found no significant relationship between SO and control ratings at the between-subject level (r = 0.20, *P* = 0.34, Figure S5A), and a positive significant relationship between SO and control ratings at the within-subject level (t = 2.55, *P* = 0.02, Figure S5B). Finally, we found no relationship between SA and control ratings for both between-subject (r = 0.31, *P* = 0.11, Figure S5C) and within-subject (t = 1.39, *P* = 0.18, Figure S5D) correlations.

#### Study II

Similarly to Study I, we found a significant positive between-subject relationship between both SO and SA (r = 0.72, *P* < 0.0001, Fig. 6C). We also found that, on a trial-by-trial basis, SO and SA correlated positively at the within-subject-level (t = 5.92, *P* < 0.0001, Fig. 6D).

The same comparisons between SO and control ratings, as well as between SA and controls ratings, were repeated. We found a positive significant relationship between SO and control ratings at the between-subject level (r = 0.62, *P* = 0.0007, Figure S5E), and a positive significant relationship between SO and control ratings at the within-subject level (t = 4.14, *P* = 0.0003, Figure S5F). Finally, we found a positive significant relationship between SA and control ratings at the between-subject level (r = 0.44, *P* = 0.02, Figure S5G) and no relationship between SA and control ratings at the within-subject level (t = 1.24, *P* = 0.23, Figure S5H).

To summarize, we found that the factors "synchrony" and "congruency" strongly modulated SO ratings, but not the factor "movement type". In addition, an increased force was associated with increased SO ratings during synchronous conditions in Study II only. SA ratings were modulated by the factors "movement type", "synchrony" and "congruency". In addition, an increased force was associated with increased SA ratings during synchronous conditions in both studies. The force positively modulated SA ratings (and SO ratings to a lesser extent) and this effect was found at the within-subject level and not at the between-subject level. Control ratings were modulated by the factors of “movement type”, "synchrony", and "congruency" in Study I only. In addition, control ratings were modulated by the force in both studies, however these effects were not confirmed by our post-hoc explorations. We found no effect for the proprioceptive drift. Finally, there was a significant positive relationship between SO and SA ratings, both at the between-subject and within-subject levels.

## Discussion

In the present study, we developed novel experimental paradigms combining robotics and virtual reality to induce altered states of SO and SA in two independent experiments. We manipulated 3 experimental factors: (1) the "type of movement" performed by the participants (active or passive); (2) the "synchrony" between the movements of the participant's hand and of the seen virtual hand (synchronous or asynchronous); and (3) the "congruency" between the hand of the participants and the seen virtual hand (right or left virtual hand moving). These experimental conditions led to a fine-grained modulation of a participant's SO and SA towards the virtual hand on a trial-by-trial basis. We examined the change in SO and SA via the experimental factors and the interaction force between the finger of the participants and the robotic device. The report of subjective ratings on a trial-by-trial basis combined with the condition-rich design allowed us to investigate in depth the complex relationship between SO and SA, both at the between-subject and within-subject levels. With these data, we could replicate the already well-studied effects of the tested experimental factors on SO and SA. The main novelties of our study are: (1) we investigated the contributions of interaction force to SO and SA; (2) we considered the shared variance associated with the experimental factors to demonstrate that SO and SA interact beyond their common multisensory basis rather than simply being co-modulated by the same experimental factors.

As supported by our results, subjective reports of SO were strongly modulated by the factor of "synchrony" and by the factor of "congruency", but not by the factor of "movement type". The present data replicates previous findings^5,6,9–11,14,21^. Classically, SO has been manipulated under static conditions, suggesting that active movements are not necessary. The present data support the idea that body ownership is mainly a perceptual phenomenon (i.e. sensory signals are at the basis of body ownership). Although this was not the case in the present data, several studies reported that SO is increased during active movements compared to passive movements^4,7,13,15^. Since active movements are not necessary for experiencing SO towards an external hand (e.g. the rubber hand illusion), this effect might be mediated through an interplay between SO and SA and might also depend on the specific procedure (e.g. duration of stimulation) and/or the type of measures used to assess SO (e.g. questionnaire or drift).

In contrast, subjective reports of SA were modulated by the factors of "type of movement", "synchrony" and by the factor of "congruency", which is consistent with previous findings^5,6,9–11,14,21^. Classically, the congruent visual structure of the controlled hand contributes mildly to SA considering that the experience of agency is not limited to bodily objects^35^ and that humans are able to feel in control of objects via indirect interactions such as response buttons. The magnitude of the effects of “congruency” reported here for SA were relatively low compared to the observed effects of “movement type” and “synchrony”, suggesting that the congruent structure, in terms of shape, of the seen hand affects SA, but to a lesser extent compared to movement generation and synchrony^5,9,10^. Cumulatively, the effects of our experimental factors associated with SO and SA are highly consistent with previous literature^4–11,13–15,21^.

The novelty of this study is that we were able to investigate whether the interaction force between the participant and the environment contributes to SO and SA and whether it might interact with other experimental factors. We found that SO was only partially modulated by the interaction force (interaction “force x synchrony” in Study II), and that there was no main effect of force on SO when comparing directly SO, SA and force in the same statistical models. Thus, our results suggest that interaction force contributes mildly to SO and this effect might be mediated through higher-order interactions between SO and SA. Indeed, SA was consistently modulated by the interaction force across both studies, and this modulation was dependent on the synchrony. In synchronous conditions, an increased interaction force predicted greater SA ratings. It is important to note that this effect was found for both passive and active conditions, as we did not find an interaction between "force" and "movement type" for SA ratings. Critically, this suggests that even during passive trials a modulation of SA by interaction force was present. Although the interaction force produced during passive trials was weaker than during active trials (see Fig. S5), the passive biomechanics of the hand at rest might produce relevant changes in interaction force that contributes to SA.

SA is often explained as the result of the comparator model^36,37^ that compares predicted and observed outcomes of actions; highest SA is associated with no difference between movement prediction and sensory feedback, while SA decreases as a function of increasing mismatch between the two. There are two possibilities to explain why a higher force would modulate the output of this system. First, it is possible that an increased force allows generating better predictions. Intuitively, exerting a stronger force under ecological conditions (i.e. no conflicts) would increase the likelihood of observing the effect of this force. If this were true, there should be a critical distinction between active and passive conditions, as there should be no prediction, or at least a different prediction, generated during passive conditions. This was not the case. The second explanation would be that the increased force generates more salient and richer sensory feedback. Indeed, the amount of force exerted would allow increasing the signal-to-noise ratio of sensory information (visual, somatosensory and proprioceptive) available to make the judgment of who is in control of the observed actions. Alternatively, there are also other possible explanations not involving the comparator model. It is possible that the subjects used a cognitive strategy to consciously identify the presence of a visuo-motor conflict by increasing the force during active conditions and by resisting the imposed movements during passive conditions. Finally, we cannot entirely exclude that this is a simple effect of arousal or saliency associated with an overall increased engagement of the motor system. Recent studies have established a link between motor attributes and subjective body experience (e.g. visuomotor interference^22^, movement kinematics^23^, motor expertise^24^, and motor accuracy^18^). To the best of our knowledge, this is the first study investigating the link between SO, SA and the interaction force between the agent and its environment.

Data from previous studies and from the present two experiments demonstrate that SO and SA are tightly linked components of subjective body experience and that they are modulated by the same experimental factors (e.g. synchrony). This is further supported by the presence of a statistical correlation between subjective reports of SO and SA (present and previous studies^10,11,21^). These results support the view that both SO and SA are components of self-consciousness generated by low-level sensorimotor processing^2,3^. However, there is accumulating evidence that SO and SA are preferably modulated by different types of low-level sensorimotor signals. For example, SO is less affected by the type of movement compared to SA (present and previous studies^4,5,13,15^). Contrastingly, SA is less affected by the congruent appearance of the owned hand compared to SO (present and previous studies^5,9,10^). In addition, the amount of temporal asynchrony differentially affects SO and SA^38,39^. Cumulatively, these results suggest that SO and SA are distinct neural processes with a certain degree of interplay^40^. The present data thoroughly demonstrate the link between SO and SA even when accounting for their common multisensory basis. This shows that, while SO and SA both depend on low-level sensorimotor signals, they still interact beyond their multisensory co-dependence. Although, we cannot disentangle between SO and SA interacting at a low sensorimotor level or at a higher cognitive level, our unique design allowed us to rule-out that SO and SA are simply co-modulated by the same experimental factors. Previous studies have also proposed interactions between SO and SA at various levels of sensorimotor hierarchy^4,7,8,13,14,41^.

Our unique experimental design allowed us to test whether SO and SA modulation is explained by variability at the population level (between-subject) or at the single subject level (within-subject). Overall, our results suggested that the contributions of interaction force to SO and SA occurred at the single-subject level and not at the population level. This shows that participants produced/perceived stronger interaction force in some trials compared to others, which led to variations in SO and SA, rather than some participants producing/perceiving overall stronger interaction force compared to other subjects. This might suggest that the highlighted effects of interaction force are associated with endogenous brain mechanisms (e.g. the comparator model), rather than population-level biases (e.g. suggestibility^42^).

Unexpectedly, our experimental factors also modulated the control rating ("I felt as if my real hand was disappearing"). Classically, disembodiment is not systematically quantified during bodily illusions. However, several studies have reported that during illusory ownership, participants can experience a less vivid sensation of their own body^43–45^. Quantitatively, disembodiment is usually less strong than the experience of ownership towards the virtual (or fake) hand^42^. Anecdotally, in the present study, a small number of participants spontaneously reported "experiencing more vividly the virtual hand than their own", which can be interpreted as a disembodiment of their real hand and might explain these unexpected findings. The stronger relationship between SO and control ratings in our results compared to SA and control ratings further support the hypothesis that the control rating indeed captured disembodiment.

The absence of effects associated with the proprioceptive drift in our behavioral experiment (Study I) was mostly due to a large inter-trial and inter-subject variability. This could be attributed to the rather difficult and uncommon setup of the task, which required participants to establish a mapping between the virtual and the real space. In addition, it is possible that, during passive or active movements, the proprioceptive input contributes to updating the perceived hand position, which might cancel out the drift induced by the illusion. Similarly, the constant contact between the user and the device generating task-irrelevant somatosensory inputs is an additional factor that might explain the absence of proprioceptive drift.

We note that few inconsistencies regarding significant main effects and interactions are found across the two studies (e.g. the main effect of force for SA present for study II, but not study I, or the effects associated with the control ratings). Although overall the agreement between the two independent studies is quite strong, the observed discrepancies can be explained by one major difference between the two studies. Indeed, study II was conducted in MR-environment, which might affect the participant’s experience due to fatigue, discomfort, drowsiness or ambient noise and might explain the observed discrepancies.

A possible limitation of the study is that active and passive conditions were not completely identical, as active movements were performed against a rendered spring and passive movements were performed against passive finger biomechanics, resulting in smaller interaction force. We note that the latter could be considered as a weaker spring but acting in the opposite direction. For these reasons, it was not possible to fully disentangle the voluntary (central force command) and involuntary force contributions (gravity, passive biomechanics). This aspect could be addressed by including EMG recordings of the intrinsic hand muscles.

The present results might have important translational applications for the fields of robotics and neuroprosthetics. An important challenge in these fields is to develop devices that can be seamlessly interfaced with the user's nervous system, with the goal of allowing them to feel and control the device as part of their own body. Strategies to provide natural force control and feedback during prosthetic usage are currently being researched^46–48^, but understanding the relationship between motor control, sensory feedback, and subjective body experience (e.g. SO and SA) might further improve usage experience and prosthesis acceptance for users^49^.

To conclude, we combined robotics and virtual reality to investigate the contribution of motor engagement (i.e. the interaction force between the user and the environment) to SO and SA, and whether interaction force is associated with well-established modulators of SO and SA. In line with previous work, our behavioral results further support the view that SO and SA are distinct processes both relying on low-level multisensory and sensorimotor signals. Furthermore, we demonstrated a novel finding by showing that the interaction force between the agent and its environment contributes to SA, but to a lesser extent to SO. These investigations have been carried out specifically in the context of hand self-consciousness. It remains unclear whether such processes for other body parts such as face or trunk rely on the same mechanisms or whether there exist body-part specific mechanisms of SO and SA.

## Methods

We conducted two independent studies with identical experimental design in two different groups of participants.

### Study I

Study I was conceived as a purely behavioral experiment aiming at investigating the link between SO, SA and interaction force.

#### Participants

For Study I, a group of 27 naive, right-handed healthy participants (5 females) aged between 18 and 39 years old (mean ± std: 24.3 ± 5.2 years) were recruited. All subjects gave written informed consent. All procedures were approved by the Ethics Committee of ETH Zurich (EK 2013-N-81), and the study was conducted in accordance with the Declaration of Helsinki.

#### Robotic manipulandum

**Figure 7:**
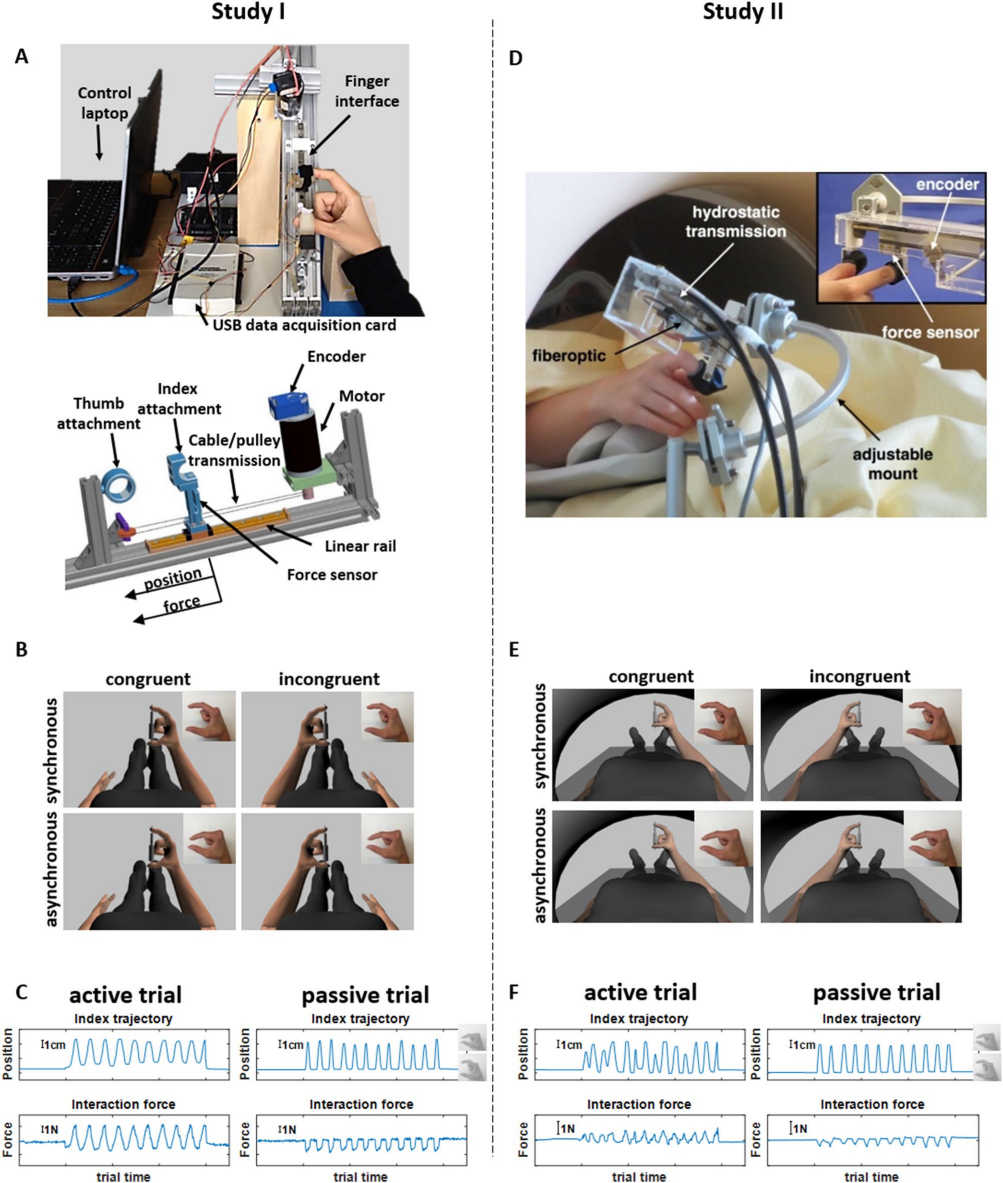
Experimental setups. (**A**) The experimental setup of Study I consists of a robotic finger manipulandum controlled with LabVIEW via a USB data acquisition card. The device is placed over the abdomen of the participant on a support structure. The right hand of the subject rests on a wooden block. The participant wears a head-mounted display (not shown). The robotic device allows performing a pinching movement either actively or passively by moving the index finger against the thumb. It is composed of a fixed thumb socket, a moveable index finger socket attached to a linear rail, a motor to actuate the index finger socket via a cable/pulley transmission, an angular position encoder and a force sensor. (**B**) A virtual environment displays an avatar performing a pinching movement. The synchrony (synchronous vs asynchronous) and the congruency (left vs right virtual hand moving) can be manipulated under computer control. (**C**) Examples of position and force traces for an active trial (i.e. the subject moves) and a passive trial (i.e. the robot moves). (**D**) The experimental setup of Study II consisted of an MR-compatible robot controlled with LabVIEW. The output of the device is attached to the scanner bed with an adjustable mount. The forearm of the subject rests on foam pads. The participant wears MR-compatible goggles (not shown). The robotic device is equipped with a fiber-optic force sensor and an electro-optical encoder, and allows performing a pinching movement either actively or passively by moving the index finger against the thumb. A DC motor actuates a linear drive through a spindle, to which a hydraulic piston was fixed and remotely controlled the slave module inside the scanner room through a hydrostatic transmission. (**E**) A virtual environment displays an avatar performing a pinching movement. The synchrony (synchronous vs asynchronous) and the congruency (left vs right virtual hand moving) can be manipulated under computer control. (**F**) Examples of position and force traces for an active trial (i.e. the subject moves) and a passive trial (i.e. the robot moves).

The robotic device was composed of a fixed thumb socket and a mobile index finger socket attached to a passive linear rail (1 degree-of-freedom) and connected to a DC motor (Maxon DC motor RE40 150 Watt, Maxon motor AG, Sachseln, Switzerland) via a cable/pulley transmission (Fig. 7A). An optical encoder (HEDM-5500, Agilent Technologies) was used to track the rotation of the motor shaft. A force sensor (CentoNewton, 40 N, Institute of production and robotics, EPFL) placed below the end effector allowed monitoring the interaction force between the subject and the robotic device. The rest of the hardware consisted of a data acquisition card (DAQ M Series NI USB-621x, National Instruments Corporation, Austin, USA) and a linear amplifier (ESCON 50/5 Servo Controller P/N 409,510, Maxon motor AG, Sachseln, Switzerland). The controller software was designed with LabVIEW (National Instruments Corporation). The virtual environment (Fig. 7B) was designed using Poser9 (SmithMicro, Inc.) and rendered using an OPEN-GL based software (ExpyVR, http://lnco.epfl.ch/expyvr). A UDP connection was implemented for communication between the controller and the rendering software.

The robotic system was designed to allow the user to perform active and passive finger pinching movements. During active movements, a virtual spring was simulated (impedance control, stiffness and damping: k = 1 [N/cm] and c = 0.14 [N*s/cm], note that the damping component was included to improve the stability of the controller) and the user could perform pinching movements by actively approaching the index finger towards the opposing thumb.

The rendering of a virtual spring was preferred over a transparent movement to better mimic the ecological sensation of pinching a compressible object (as displayed in the virtual environment). During passive movements, a preplanned position trajectory was imposed to the user's index finger using a proportional-derivative position controller (position control, k_p_ = 5.7, k_d_ = 0.21). The sequence consisted of successive pinching movements towards the fixed thumb following a sinusoidal trajectory with variable frequencies, amplitudes and interstimulus intervals (ISI) between two successive pinching movements (frequency ranged between 0.6 and 0.8 Hz, amplitude ranged between 66 and 100% of maximum aperture, ISI ranged between 900 and 1370 ms). During passive stimulation, each trial consisted of 12 pinching movements. During active movements, the number of pinching movements was 12.2 ± 6.1 (mean ± std). Examples of position and force traces measured during an active trial and a passive trial are shown in Fig. 7C. Variability in the sinusoidal trajectories was included to mimic the characteristics of human movements.

#### Experimental setup

Participants lay in supine position on a bed with a head-mounted display fixed on the head (Oculus DK1, Oculus VR, LLC, Menlo Park, CA, USA; resolution: 1280 × 800 pixels at 60 Hz). The right hand, fixed to the robot, was positioned 15 cm to the right of the body midline (Fig. 7A). The robot was placed over the abdomen on a support structure. The right arm was rested on a wooden support to guarantee the participant's comfort. The left arm was rested on the bed along the body and the left hand was used to control response buttons. In the HMD, a virtual environment was displayed and showed a first-person perspective with a direct view on the right hand and the body of the avatar. The virtual hand was positioned at the body midline of the avatar and was holding a compressible piston (Fig. 7B). A spatial offset between the real and virtual hands was introduced to measure a potential proprioceptive drift towards the virtual hand. The avatar was matched according to each participant's gender and skin color.

#### Experimental conditions

During each experimental trial, participants performed pinching movements for 30 s with their right hand while observing the virtual hand also performing pinching movements (Fig. 7A-B). There was a total of 8 experimental conditions with 3 factors being manipulated (2 × 2 x 2 design). First, the type of movement produced by the participant's hand could be active or passive (factor of "movement type"). Second, the virtual movements were either synchronous or asynchronous with the movements of the participant's hand (factor of "synchrony"). During asynchronous conditions, the virtual avatar performed a different preplanned sequence of pinching movements, leading to both spatial and temporal mismatch between the movements of the participant's hand and of the avatar's hand. Finally, the virtual moving hand was either the right or the left hand of the avatar, while participants always performed the movement with their right hand (factor of "congruency"). Each condition was repeated 8 times, leading to a total of 64 trials divided into 4 runs of approximately 15 min each. The 4 runs consisted of 2 runs with active movements and 2 runs with passive movements. The order of the runs was pseudo-randomized across participants and the order of condition presentation within the same run was randomized.

#### Experimental measures

Participant’s responses were collected using pushbuttons controlled with the left hand. To measure a potential proprioceptive drift induced by the stimulation, participants conducted a hand localization task by reporting the perceived position of their right hand using a virtual horizontal ruler displayed in the HDM (Figure S1A). This localization task was performed at the beginning and at the end of each trial, and consisted in aligning the ruler with the perceived position of the participant's right hand along the displayed horizontal line. During the localization task, participants were instructed to refrain from moving their head. The proprioceptive drift was measured as the difference in localization at the beginning and at the end of each trial.

Following each stimulation period (and after the localization task), participants rated 3 statements aiming at evaluating their subjective SO towards the virtual hand and SA over the virtual movement, as well as a control statement.

- Ownership rating: "I felt as if the virtual hand was my own hand"
- Agency rating: "I felt as if I was producing the virtual hand movements"
- Control rating: "I felt as if my real hand was disappearing"

A virtual ruler was used by the participants to rate these items on a Likert scale ranging from 1 to 7 (Figure S1B), with 1 corresponding to "totally disagree" and 7 corresponding to "totally agree". After the subjective ratings and before starting the next stimulation period, there was a rest period of 3 s. During the localization task, subjective ratings and rest period, the robot was set in stand-by mode, preventing movements of the participant's right hand.

#### Experimental procedure

Participants were first given an explanatory session to familiarize with the robotic device and the HMD. A demonstration of active and passive movements with the robotic manipulandum was presented. Subjects received instructions regarding the type of pinching movements to be performed during the experiments. For passive movements, participants were trained to relax their fingers and avoid generating any voluntary force on the robot. For active movements, participants were trained to perform smooth and uninterrupted pinching movements from start to end (a complete closing and opening movement). In particular, they were asked to mark a pause between each successive pinching cycle and to try, as best as possible, to maintain a regular pace similar to the one previously demonstrated with passive movements (between 0.6 and 0.8 Hz approximately). An adapted and comfortable maximal aperture was set for each participant, corresponding to the equilibrium point of the virtual spring.

After the familiarization with the setup, the experimenter introduced the different experimental measures (proprioceptive drift, ownership rating, agency rating and control rating) to ensure the participant's understanding and demonstrated how to use the response buttons. For the localization task, participants could familiarize with the digital ruler to align it with the perceived position of their hand.

Participants were instructed to fix and maintain their attention on the virtual hand during the 30 s stimulation and to refrain from moving their head. Rather than including an attention control task, we considered that the proprioceptive task and the rating of items every 30 s were sufficient to ensure the participant's arousal and attention. An experimenter monitored whether participants were indeed conducting the tasks as instructed. Before the beginning of the experiment, participants were fitted with black clothes (pants, socks and a t-shirt) to match the ones of the avatar displayed in the HMD.

### Study II

Study II was conceived as an fMRI experiment to study the neural correlates of SO and SA. fMRI results will be presented in a different publication. The design was the same as for Study I, except that we did not measure the proprioceptive drift for time constraints. In addition, we used MR-compatible equipment for the robotic and virtual reality setup.

#### Participants

A group of 26 naive and right-handed healthy participants (14 females) aged between 18 and 35 years old (mean ± std: 25.5 ± 4.3 years) were recruited for the study. All subjects gave written informed consent. All procedures were approved by the Cantonal Ethics Committee, Department of Health of the Canton Zurich (KEK2010-0190), and the study was conducted in accordance with the Declaration of Helsinki.

#### MR-compatible robotic manipulandum

The robotic device was an MR-compatible homolog of the device used in the behavioral experiment. It was also composed of a fixed thumb socket and a mobile index socket. A DC motor (Maxon RE40, Switzerland) actuated a linear drive through a spindle, to which a hydraulic piston was fixed. This piston remotely actuated the slave module inside the scanner room through a hydrostatic transmission^50^ (Fig. 7D). The position of the slave module was measured with an electro-optical encoder (LM 12CPMM, Encoder Technology, USA, resolution = 0.08 mm). Interaction forces during movements were measured with an MR-compatible custom-built fiber-optic force sensor^50^. This setup was tested and used during previous studies^51,52^. The controller software was designed with LabVIEW (National Instruments Corporation). The virtual environment (Fig. 7E) was designed using Poser9 software (SmithMicro, Inc.) and rendered using an OPEN-GL based software (ExpyVR, http://lnco.epfl.ch/expyvr). A UDP connection was implemented to communicate between the controller and the rendering software.

Similarly to Study I, the robotic system allowed the user to perform active and passive pinching movements. During active movements, a virtual spring was rendered (position control, stiffness: k = 0.25 [N/cm]) to simulate a compressible object and the user could perform pinching movements by actively approaching the index towards the opposing thumb. During passive movements, a preplanned sequence was imposed to the user's index using a proportional-integral-derivative controller (position control, K_p_ = 80, K_i_ = 1.2, K_d_ = 1.4). The same preplanned trajectories were used as in Study I. During passive stimulation, each trial consisted of 12 pinching movements. During active movements, the number of pinching movements was 10.8 ± 3.5 (mean ± std). Examples of position and force traces measured during an active trial and a passive trial are shown in Fig. 7F.

#### Experimental setup

Participants were laid in supine position on the scanner bed. The right hand was fixed to the robot, which was mounted on an adjustable aluminum structure (Fig. 7D). The right arm was supported by foam pads to guarantee the participant's comfort. The left arm was rested on the scanner bed along the body and the left hand was used to control MR-compatible response buttons. MR-compatible goggles (manufacturer: Resonance Technology, Inc., Northridge, CA, USA; resolution: 800 × 600 pixels at 60 Hz) were used to display a virtual environment showing a first-person perspective with a direct view on the right hand and the body of the avatar. The virtual hand was positioned at the body midline of the avatar and was holding a compressible piston (Fig. 7E). The avatar was matched according to participant's gender and skin color.

#### Experimental conditions

As in Sect. 4.1.4.

#### Experimental measures

As in Sect. 4.1.5. The proprioceptive drift was not measured during the fMRI experiment due to time constraints.

#### Experimental procedure

As in Sect. 4.1.6.

### Statistical analyses

#### Data preprocessing

For Study I, the results of the hand localization task (i.e. proprioceptive drift) were transformed into physical distances in cm by taking into account the precise distance between the participant's right hand and body midline.

A measure of the “amount” of force produced during each trial was computed as follows (Fig. 7C and F). The force recordings were first downsampled from 200 to 10 Hz and filtered using a lowpass Chebyshev filter (order = 8). Second, each trial was baseline corrected by subtracting the mean force measured during the preceding rest period. Finally, the absolute values of the difference between each pair of consecutive datapoints were summed to obtain a measure of the “amount” of force produced during each trial. This measure corresponds to the integral of the absolute force during each trial.

#### Linear Mixed Models with interaction force as covariate

To investigate the contribution of the interaction force to the experimental measures, subjective ratings (Studies I and II) and proprioceptive drifts (Study I) were analyzed separately using linear mixed models^53,54^. We hypothesized that interaction force will have a strong impact on SA due to the role of interaction force in motor control and efferent mechanisms, and less impact on SO, which is considered a rather perceptual phenomenon.

The “Force” measure was used as covariate in the linear mixed models. All experimental trials were used as observations (64 observations per subject). Separate linear mixed models were computed for each measure (total of 7 linear mixed models). Each model consisted of experimental, fixed factors ("movement type", "synchrony" and "congruency"), a random factor (“subject”) with random intercept, and a covariate, the interaction force between the participant and the robotic device ("force"). P-values were obtained by likelihood ratio tests and degrees of freedom were approximated using the Kenward-Roger method.

For significant main effects associated with the experimental factors, we graphically presented data for individual subjects along with the average across subjects. For each subject, ratings were averaged across all trials of the same level to obtain one data point per subject for each level, e.g. one average synchronous rating and one average asynchronous rating for each subject for a main effect of “synchrony”. We also report the mean difference (MD) and standard-deviation (SD) for these data.

For significant effects associated with the force covariate, we conducted post-hoc analyses to further investigate the respective contributions of between-subject and within-subject variability. For between-subject variability, ratings and force were averaged across all trials to obtain one data point per subject and we computed a linear regression across subjects with ratings as observed variable and force as predicting variable. This aimed at testing whether between-subject variations in force (i.e. some subject produced/perceived overall stronger interaction force compared to other subjects) were associated with variations in SO and SA. For within-subject variability, we computed separately for each subject a linear regression across all trials with ratings as observed variable and force as predicting variable. The obtained regression coefficients were tested with a one sample t-test in case of a main effect of “force” and with a paired t-test in case of an interaction between “force” and an experimental factor (e.g. “force x synchrony”). This aimed at testing whether variations in interaction force across trials of the same subject (i.e. the same subject produced/perceived stronger interaction force in some trials compared to other trials) were associated with variations in SO and SA. The rationale to investigate separately between- and within-subject variability is to gain insights into the underlying mechanisms, in particular significant between-subject post-hoc comparisons would point towards population level effects and individuals traits, while significant within-subject post-hoc comparisons would point towards endogenous neural mechanisms.

Finally, to provide direct comparisons between SO, SA and force, we computed two additional linear mixed models: 1) a model with SO as observed variable and with SA and force as explanatory variables; 2) a model with SA as observed variable and with SO and force as explanatory variables. These models included the main effects, the interaction term and a random factor (“subject”) with random intercept. As previously, p-values were obtained by likelihood ratio tests and degrees of freedom were approximated using the Kenward-Roger method.

Each computed linear mixed model was considered an independent analysis aiming at testing a specific and precise hypothesis. For this reason, we did not apply a correction for multiple comparisons to our statistical results for the number of linear mixed models computed.

#### Linear Mixed Models with interaction force as observed variable

We also conducted a supplementary analysis using linear mixed models with the interaction force as observed variable and with "movement type", "synchrony" and "congruency" as fixed effects and a random effect “subject” with random intercept. We expected a strong effect of the “movement type” factor on the force with increased force during active trials.

#### Correlations between SO and SA

The inclusion of several different modulators of SO and SA in our design allowed accounting for the variance associated with these modulators to investigate a deeper relationship between SO and SA. To investigate this relationship, while accounting for the common multisensory basis of SO and SA, we regressed out from SO and SA ratings the variance associated with the experimental factors of movement type, synchrony and congruency. We hypothesized that the relationship between SO and SA goes beyond their common multisensory basis and that we would find a strong relationship between SO and SA even after regressing out the shared variance explained by the experimental factors. As previously, we conducted separate analyses to assess the respective contributions of between- and within-subject variability.

First, to regress out the shared variance between SO and SA associated with the experimental factors, we computed linear mixed models for SO and SA with "movement type", "synchrony" and "congruency" as fixed effects and a random effect “subject” with random intercept. The residuals of these models were used to compute between-subject and within-subject correlations between SO and SA. For between-subject correlations, the overall average SO and SA ratings for each subject were computed and a correlation was computed across subjects. For within-subject correlations, a correlation between SO and SA ratings across all trials was computed separately for each subject. These correlation values were then transformed to normal values using the Fischer transform and used to compute a one-sample t-test. These analyses were replicated to investigate possible correlations between SO ratings and the control ratings, as well as between SA ratings and the control ratings.

### Ethics approval statement

Research was conducted under the approval of the Ethics Committee of ETH Zurich (Study I, EK 2013-N-81) and the Cantonal Ethics Committee, Department of Health of the Canton Zurich (study II, KEK2010-0190). Written informed consent was obtained from all experimental subjects.

## Supplementary Information

Supplementary Information.

## Data Availability

The datasets generated during and/or analyzed during the current study are available from the corresponding author on reasonable request.
