# Supplementary material for: Contribution of interaction force to the sense of hand ownership and the sense of hand agency
Source: Sci Rep. 2021 Sep 10;11:18069. doi: 10.1038/s41598-021-97540-9 (PMC8433290; doi:10.1038/s41598-021-97540-9)
Supplement: Supplementary file 1 — Supplementary Information. [file 41598_2021_97540_MOESM1_ESM.pdf]

Contribution of interaction force to the sense of hand ownership and the sense of hand agency

Michel Akselrod[1,2,3,4,*], Bogdan Vigaru[1], Julio Duenas[1], Roberto Martuzzi[4,5], James Sulzer[6,1], Andrea Serino[2], Olaf Blanke[4,7] and Roger Gassert[1,*]

[1] Rehabilitation Engineering Laboratory, ETH Zurich, Zurich, Switzerland
[2] MySpace Laboratory, University Hospital, Lausanne, Switzerland
[3] Cognition, Motion and Neuroscience Unit, Minded Programme, Fondazione Istituto Italiano di Tecnologia, Genova, Italy
[4] Laboratory of Cognitive Neuroscience, Federal Institute of Technology of Lausanne (EPFL), Lausanne, Switzerland
[5] Fondation Campus Biotech Geneva, Geneva, Switzerland
[6] Rewire Laboratory, University of Texas, Austin, USA
[7] Department of Neurology, University Hospital of Geneva, Geneva Switzerland

Corresponding authors:

Michel Akselrod
Laboratory MySpace
Department of Clinical Neuroscience
University Hospital of Lausanne (CHUV)
Avenue Beaumont, Pavillon 4
CH-1011 Lausanne, Switzerland
michel.akselrod@gmail.com

Supplementary materials

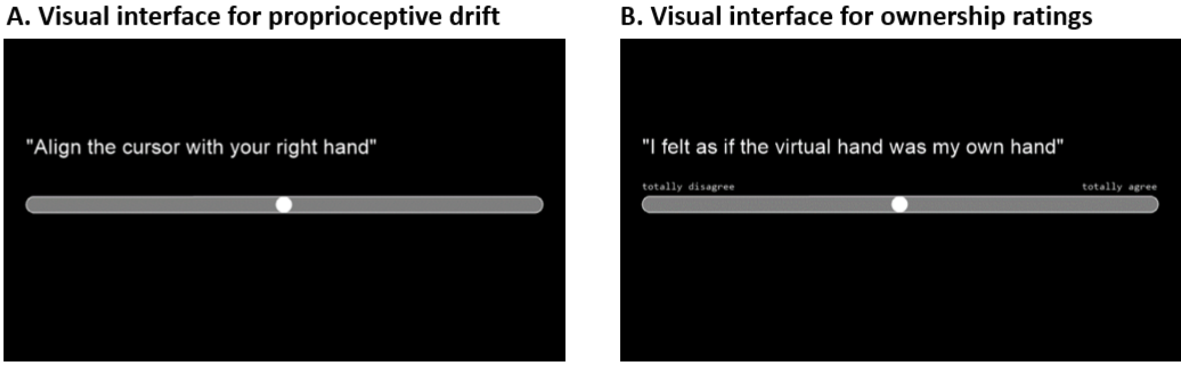

Figure S1. Visual interfaces for proprioceptive drift and ratings. *A) Interface for proprioceptive drift assessment. A virtual horizontal ruler was controlled by the participant's left hand using buttons. B) Interface for ownership ratings. A virtual horizontal ruler was controlled by the participant's left hand using buttons. Similar interfaces were used for agency ratings and control ratings, as well as for Study I and Study II.*

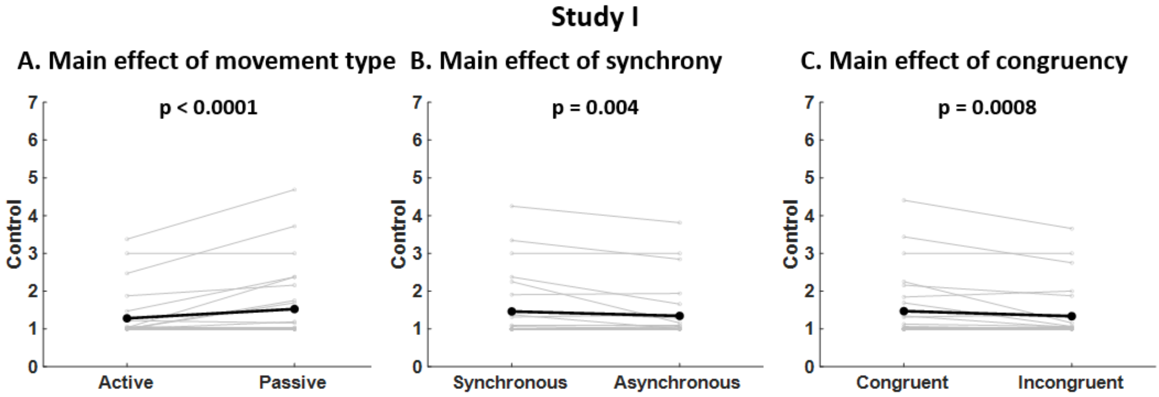

Figure S2. Control ratings (Study I). *A) Control ratings for active and passive conditions are shown for Study I. B) Control ratings for synchronous and asynchronous conditions are shown for Study I. C) Control ratings for congruent and incongruent conditions are shown for Study I. For each panel, data plotted in grey represent the mean rating for individual subjects and data plotted in black represents the mean rating across subjects. The p-values correspond to the main effects reported in Table 1. No significant main effects were found for Study II.*

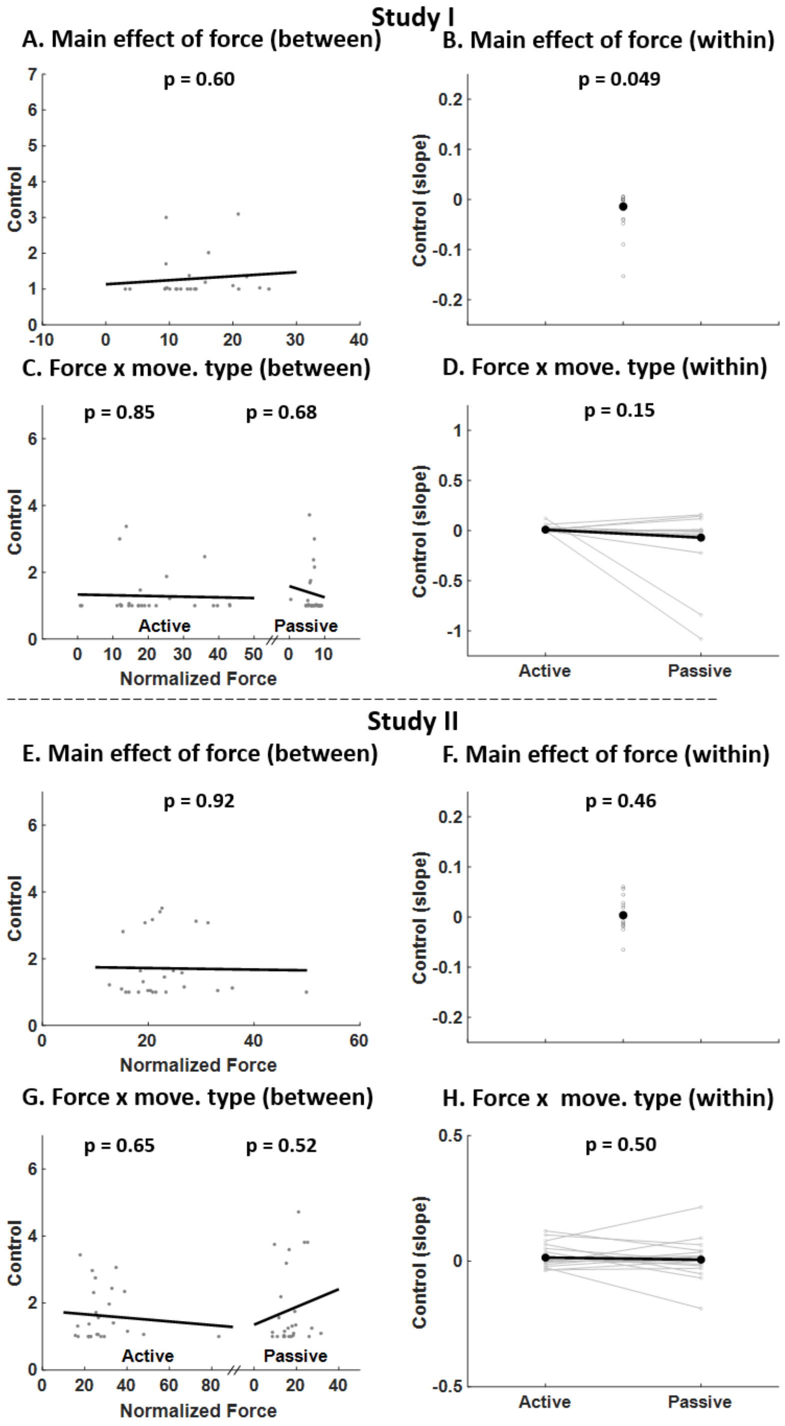

Figure S3. Control ratings - post-hoc comparisons for main effect of "force" and interaction "force x synchrony" (Studies I and II). *A-E) Between-subject regressions between control ratings and force are shown. Data points plotted in grey correspond to the mean control rating and mean force across all trials for each subject. The black lines correspond to the between-subject regression line. The non-significant regressions suggest that the main effects of "force" (Table 1, Studies I and II) are not explained by between-subject variability. B-F) Within-subject regression coefficients between control ratings and force are shown. For each subject, a regression between control ratings and force for all trials was computed and a paired t-test was computed between the regression coefficients. Data points plotted in grey correspond to individual regression coefficients and data points plotted in black represent the mean coefficients across subjects. The non-significant one-sample t-tests suggest that the main effects of "force" (Table 1, Studies I and II) are not explained by within-subject variability. C-G) Post-hoc comparisons as in A-E to evaluate the contribution of between-subject variability to the interaction "force x synchrony". The non-significant regressions suggest that the interactions "force x synchrony" (Table 1, Studies I and II) are not explained by between-subject variability. D-H) Post-hoc comparisons as in B-F to evaluate the contribution of within-subject variability to the interaction "force x synchrony". The non-significant paired t-tests between synchronous and asynchronous regression coefficients suggest that the interactions "force x synchrony" (Table 1, Studies I and II) are not explained by within-subject variability.*

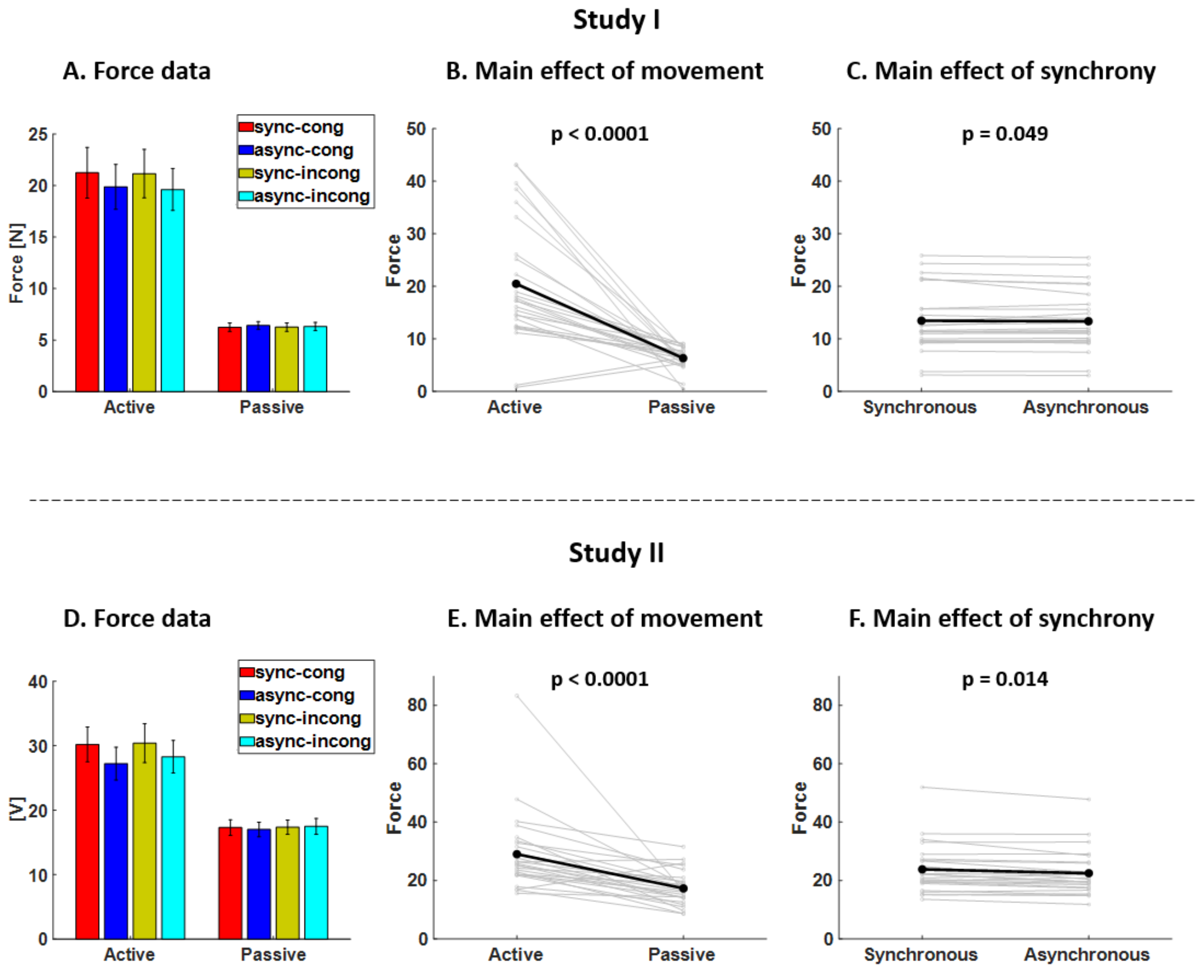

Figure S4. Force data. *A-D) Force data for Studies I and II. The 8 experimental conditions follow a 2x2x2 design: active/passive x synchronous/asynchronous x congruent/incongruent. Error bars represent the standard error of the mean. B-E) Force data for active and passive conditions are shown for Studies I and II. C-F) Force data for synchronous and asynchronous conditions are shown for Studies I and II. For panels B-C-E-F, data plotted in grey represent the mean force for individual subjects and data plotted in black represents the mean force across subjects. The p-values correspond to the main effects reported in Table S1.*

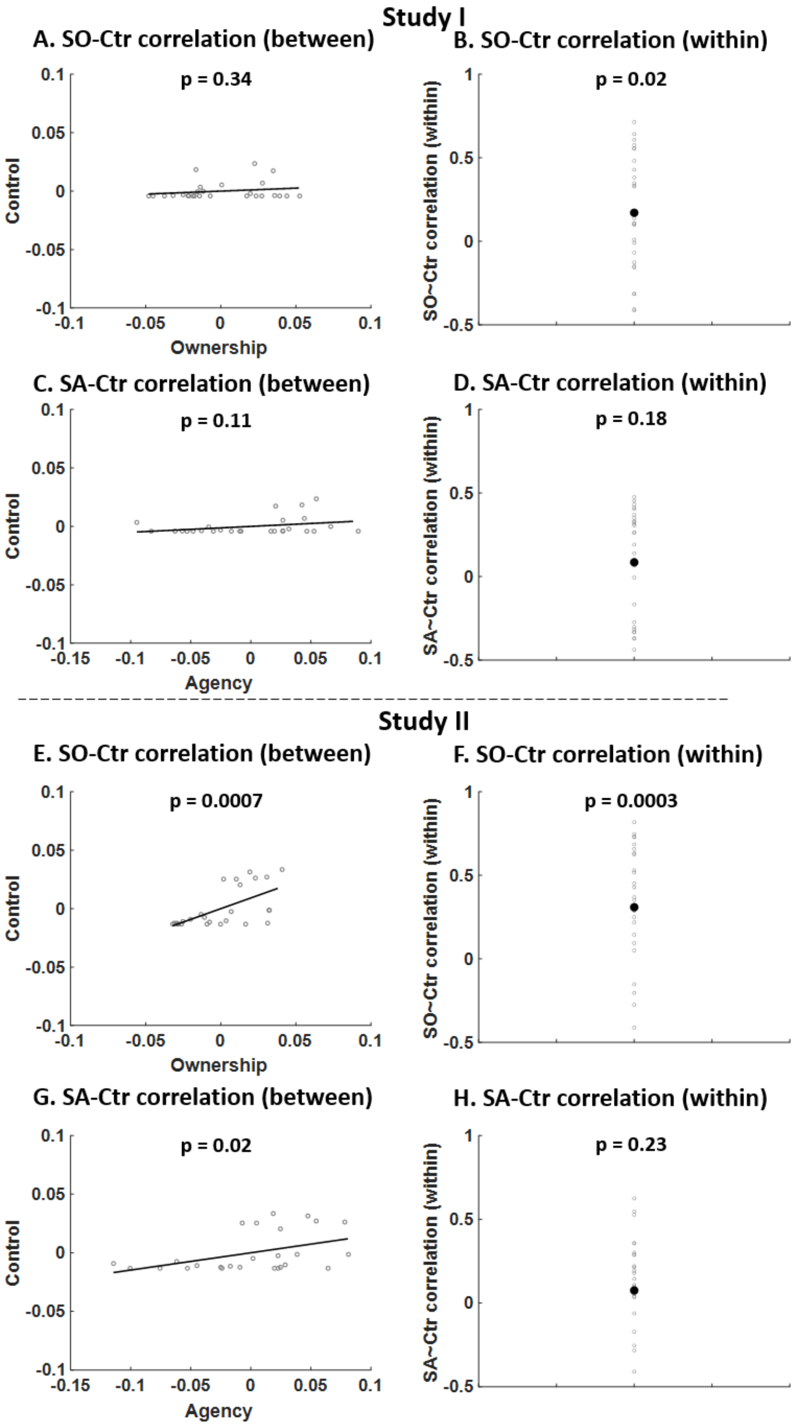

Figure S5. Control ratings - post-hoc comparisons for main effect of "force" and interaction "force x synchrony" (Studies I and II). *A-E) Between-subject regressions between control ratings and force are shown. Data points plotted in grey correspond to the mean control rating and mean force across all trials for each subject. The black lines correspond to the between-subject regression line. The non-significant regressions suggest that the main effects of "force" (Table 1, Studies I and II) are not explained by between-subject variability. B-F) Within-subject regression coefficients between control ratings and force are shown. For each subject, a regression between control ratings and force for all trials was computed and a paired t-test was computed between the regression coefficients. Data points plotted in grey correspond to individual regression coefficients and data points plotted in black represent the mean coefficients across subjects. The non-significant one-sample t-tests suggest that the main effects of "force" (Table 1, Studies I and II) are not explained by within-subject variability. C-G) Post-hoc comparisons as in A-E to evaluate the contribution of between-subject variability to the interaction "force x synchrony". The non-significant regressions suggest that the interactions "force x synchrony" (Table 1, Studies I and II) are not explained by between-subject variability. D-H) Post-hoc comparisons as in B-F to evaluate the contribution of within-subject variability to the interaction "force x synchrony". The non-significant paired t-tests between synchronous and asynchronous regression coefficients suggest that the interactions "force x synchrony" (Table 1, Studies I and II) are not explained by within-subject variability.*

Study I (n=27)

	OWNERSHIP		
effect	d.o.f.	T-statistic	p-value
AGENCY	1675	19.63	**<0.0001**
FORCE	1670	-0.51	0.61
AGENCY*FORCE	1663	-3.48	**0.0005**

	AGENCY		
effect	d.o.f.	T-statistic	p-value
OWN	1630	17.48	**<0.0001**
FORCE	1677	4.09	**<0.0001**
OWN*FORCE	1680	1.83	0.07

Study II (n=26)

	OWNERSHIP		
effect	d.o.f.	T-statistic	p-value
AGENCY	1647	14.09	**<0.0001**
FORCE	1652	0.29	0.77
AGENCY*FORCE	1642	-0.16	0.87

	AGENCY		
effect	d.o.f.	T-statistic	p-value
OWN	1640	14.18	**<0.0001**
FORCE	1626	2.63	**0.009**
OWN*FORCE	1664	0.84	0.40

Table S1. Statistical results for the direct comparisons between SO, SA and interaction force. *The statistical results of the linear mixed models for the direct comparisons between SO, SA and interaction force are presented for Study I (top panel) and for Study II (bottom panel). SO ratings are is modelled as a function of SA, the force and the interaction between the two. SA ratings are modelled as a function of SO, the force and the interaction between the two.*

Study I (n=27)

	Force		
effect	d.o.f	T-statistic	p-value
MOVE	1654	38.72	**<0.0001**
SYNC	1653	1.97	**0.049**
CONG	1653	0.31	0.76

Study II (n=26)

	Force		
effect	d.o.f	T-statistic	p-value
MOVE	1638	21.97	**<0.0001**
SYNC	1638	2.46	**0.014**
CONG	1638	-0.84	0.40

Table S2. Statistical results for Force data. *The statistical results of the linear mixed models for Force data are presented for Study I (top panel) and for Study II (bottom panel). Force data is modelled as a function of the experimental factors ("movement type", "synchrony" and "congruency").*
